# ﻿*Gesneriatuberifera* (Gesneriaceae), a new lithophytic species from the Sierra de Bahoruco, Barahona Peninsula of southern Hispaniola (Dominican Republic)

**DOI:** 10.3897/phytokeys.235.110476

**Published:** 2023-11-10

**Authors:** John L. Clark, Teodoro Clase

**Affiliations:** 1 Marie Selby Botanical Gardens, 1534 Mound St., Sarasota, FL 34236 USA Marie Selby Botanical Gardens Sarasota United States of America; 2 El Jardín Botánico Nacional, Av. República de Colombia esq. Av. Los Próceres, Sector los Altos de Gala, Santo Domingo, D.N. Dominican Republic El Jardín Botánico Nacional Santo Domingo Dominican Republic

**Keywords:** Biodiversity, Gesneriinae, Sierra de Bahoruco, systematics, taxonomy, tuber

## Abstract

A narrowly endemic new species of *Gesneria* is described from the Sierra de Bahoruco in the Dominican Republic’s Barahona Peninsula of southern Hispaniola. *Gesneriatuberifera* J.L.Clark & T.Clase, **sp. nov.** differs from all other congeners by the presence of a tuber and tubular red corollas with yellow lobes. Images and a discussion are provided to summarize the presence of tubers in other Gesneriaceae and differentiate *Gesneriatuberifera* from congeners that share a lithophytic habit. Based on IUCN guidelines, a preliminary conservation status of Endangered (EN) is assessed for *G.tuberifera*.

## Abstract (es)

Se describe una nueva especie de *Gesneria* endémica de la Sierra de Bahoruco en la península de Barahona en la República Dominicana, en el sur de La Española. *Gesneriatuberifera* J.L.Clark & T.Clase, **sp. nov.** se diferencia de todos los demás congéneres por la presencia de un tubérculo y corolas rojas tubulares con lóbulos amarillos. Se proporcionan imágenes y una discusión para resumir la presencia de tubérculos en otras Gesneriaceae y diferenciar a *Gesneriatuberifera* de otros congéneres que comparten un hábito litófito. Con base en las pautas de la UICN, se evalúa un estado de conservación preliminar de En Peligro (EN) para *G.tuberifera*.

## ﻿Introduction

The flowering plant family Gesneriaceae, with over 3400 species and 150+ genera (Weber 2004; Weber et al. 2013), is in the order Lamiales. The family is divided into three subfamilies and seven tribes (Weber et al. 2013, 2020), each of which represent monophyletic lineages (Ogutcen et al. 2021). Most Neotropical members are in the subfamily Gesnerioideae, which is represented by 1200+ species and 77 genera (Clark et al. 2020). *Gesneria* L. is classified in the tribe Gesnerieae and subtribe Gesneriinae Oerst. (Weber et al. 2013, 2020).

The subtribe Gesneriinae is strongly supported as a monophyletic clade that is mostly Caribbean and includes the following three genera: *Gesneria*, *Pheidonocarpa* L.E.Skog, and *Rhytidophyllum* Mart. *Pheidonocarpa* has one species with a subspecies endemic to Cuba and a second subspecies endemic to Jamaica. *Rhytidophyllum* is mostly endemic to the Caribbean islands, with the exception of two species in northern South America. *Gesneria* is entirely endemic to the Caribbean region and was most recently monographed by Skog (1976). Updated circumscriptions to several broadly defined species in Skog (1976) were re-circumscribed in publications by Clark et al. (2019), Lambert et al. (2017), and Joly et al. (2023). The addition of *Gesneriatuberifera* brings the total species diversity of *Gesneria* to 63 or 73 taxa when including infraspecific ranks.

*Gesneria* is broadly characterized by alternate leaves, an inferior or sub-inferior ovary, and glabrous filaments that are substantially free (adnate at base only) from the corolla tube. The habit for *Gesneria* is variable, but most species are perennial subshrubs (lithophytes or terrestrial) or lithophytes with leaves in a rosette. The corolla shape in *Gesneria* ranges from tubular to campanulate. The base chromosome number for all members of the tribe Gesnerieae is n = 14 (Lee 1966, 1968), a character that supports the monophyly of *Gesneria*, *Pheidonocarpa* L.E.Skog, and *Rhytidophyllum*. The flowers of *Gesneria* are usually zygomorphic. *Gesneriaradiata* J.L.Clark & Cinea is a recently described species (Joly et al. 2023) and is the only species in the genus with corollas that appear radially symmetrical.

Plants were photographed in the field and subsequently pressed and dried. Specimens were deposited at the Jardín Botánico Nacional ‘’Dr. Rafael M. Moscoso’’ (**JBSD**), Marie Selby Botanical Gardens (**SEL**), United States National Herbarium (**US**), New York Botanical Garden (**NY**), Missouri Botanical Garden (**MO**), and other herbaria. Photographs were taken of live specimens in the field using a Nikon D7200 DSLR camera, Nikon 105mm lens, and Nikon SB-29s ring flash. Morphological observations and measurements were made from live collections, alcohol-preserved material, and digital images using the *ImageJ* program (Schneider et al. 2012).

The extinction risk for *Gesneriatuberifera* was assessed following the IUCN (2012) and guidelines of the IUCN Standards and Petitions Committee (2022). Observations, collection localities, and population estimates from fieldwork were considered when assessing the IUCN category. Species area of occupancy (AOO) was calculated using GeoCAT (Bachman et al. 2011) with the default setting of a 2 km^2^ grid (extent of occurrence (EOO) was not calculated because of the limited number of known populations).

## ﻿Taxonomic treatment

### ﻿ Gesneria tuberifera

Taxon classificationPlantaeLepidopteraGesneriaceae

J.L.Clark & T.Clase sp. nov.

0952BC4A-C724-5019-A0D9-CD22FDA2FE0F

urn:lsid:ipni.org:names:77330583-1

Fig. 1

#### Diagnosis.

Differs from all other *Gesneria* by the presence of a tuber. Additional characters that differentiate *G.tuberifera* from congeners is a rosette of leaves, elongate red tubular corollas with yellow lobes, and a lithophytic habit.

#### Type.

**Dominican Republic. Pedernales**: Sierra de Bahoruco, Las Mercedes, km 28 on the road Cabo Rojo–Aceitillar, Cañada La U, 18°7'13.05"N, 71°37'25.09"W, 840 m, 26 Jan 2023, *J.L. Clark & T. Clase 17279* (holotype: JBSD; isotypes: FLAS, MO, MT, NY, SEL, US).

#### Description.

Unbranched lithophyte with well-developed woody tubers to 3.5 cm in diameter, older individuals with elongate woody shoots to 30 cm long subtending a rosette of leaves, younger individuals with short shoots (<5 cm long) with leaves in a basal rosette, apex of shoots covered with dense red pilose trichomes, base of shoots glabrescent. ***Leaves*** alternate, always clustered (rosette), coriaceous, petioles 0.5–1.0 cm long, velutinous, reddish; blade cuneate to broadly obovate, 5–8.5 × 1.5–2.5 cm, base attenuate, apex acute, margin sparsely serrate with 6–10 shallow lobes that become more deeply lobed near apex, margin of leaf with evenly spaced white trichomes, abaxially light green, densely pilose along main vein, especially near base, sparsely pilose along main vein near leaf apex, adaxially dark green, glabrous, lateral veins 6–9 per side. ***Inflorescence*** reduced to a single axillary flower, pedicel uniformly red, erect to horizontal, 3–5 cm long, in the upper axils. ***Floral*** tube obconic, 2–4 × 2–3 mm, uniformly red, shallowly sulcate with five ridges. ***Calyx*** lobes five, erect, 3–5 mm long, 2 mm wide, triangular, uniformly red. **Corolla** zygomorphic, uniformly tubular to slightly constricted apically, 2.2–2.7 cm long, 1–1.5 cm wide, mostly red with yellow lobes, limb with five erect lobes, subequal, semi-orbiculate, 1–2 × 1.5–2.5 mm, entire. ***Androecium*** with four stamens, 1.9–2.5 cm long, briefly adnate to the base of the corolla tube, included; anthers oblong, 1.0–2.5 × 1.0–1.5 mm; staminode present. ***Gynoecium*** with inferior ovary, disc annular, white; immature ovary globose, mature ovary not observed (flowers protandrous). ***Fruit*** a sub-woody globose bivalved capsule, 4–6 × 3–5 mm. ***Seeds*** fusiform, striated, twisted, 0.5–1.0 × 0.3 mm, dark brown to black.

#### Phenology.

Mature flowers were documented during January, February, June, and August. Mature fruits were documented during January, February, June, and August.

#### Etymology.

**Figure 1.**
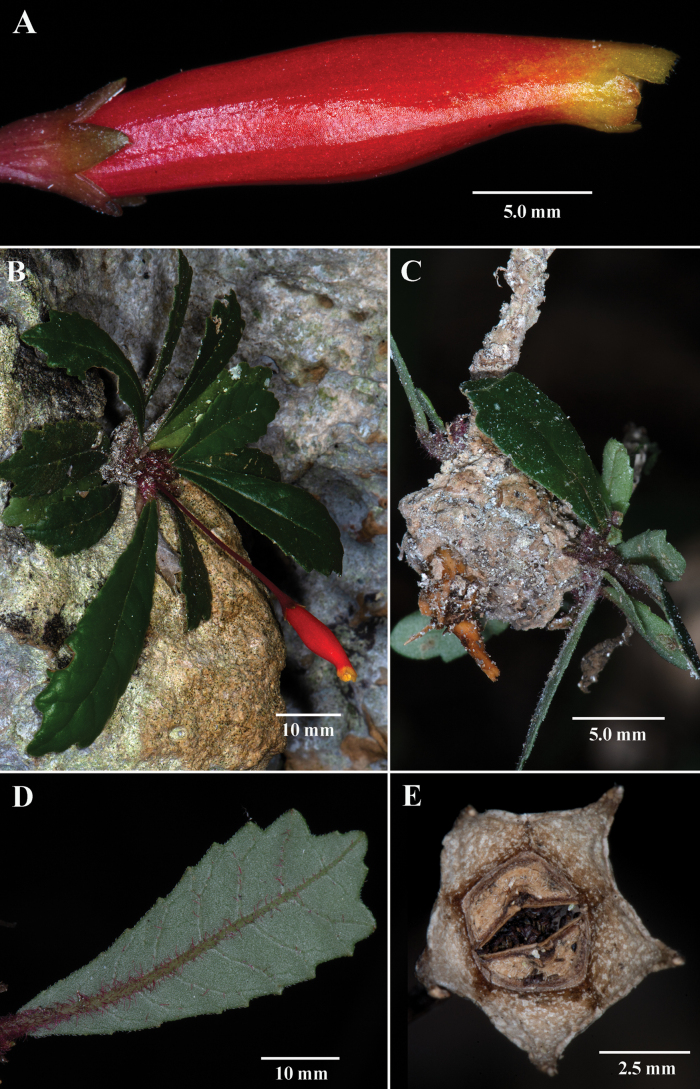
*Gesneriatuberifera* J.L.Clark & T.Clase **A** lateral view of mature flower **B** lithophytic habit **C** tuber with foliage **D** abaxial leaf surface **E** mature fruit (**A, B***J.L. Clark 17279***C***J.L. Clark 17284***E***J.L. Clark 17312*). Photos by John L. Clark.

The specific epithet means growing a tuber and is derived from the presence of a swollen stem base (Fig. 1C), a vegetative character consistent throughout the two populations observed during a 2023 field expedition to the Dominican Republic.

#### Distribution and preliminary assessment of conservation status.

*Gesneriatuberifera* is endemic to the Sierra de Bahoruco or Bahoruco mountain range in the Dominican Republic’s Barahona Peninsula of southern Hispaniola. The Sierra de Bahoruco is in the southwestern region of the Dominican Republic, adjacent to the southern border with Haiti, and encompasses the provinces of Pedernales and Barahona. Some parts of Sierra de Bahoruco comprise a national park, Sierra de Bahoruco National Park (Parque Nacional Sierra de Bahoruco). The park is recognized by the United Nations Educational, Scientific and Cultural Organization (UNESCO) as a Biosphere reserve. The forest is classified as humid forest (bosque húmedo) transitioning to broadleaf forest to pine (bosque latifoliado al pinar) in a riverside forest of a ravine (bosque ribereño de una cañada). Common woody plants in the ravine include *Cassiaspectabilis* DC. (Fabaceae), *Comocladia* P.Browne (Anacardiaceae), *Ficus* L. sp. (Moraceae), *Mastichodendron* sp. (Engl.) H.J.Lam (Sapotaceae), *Ocotea* sp. Aubl. (Laureaceae), *Oxandra* sp. A.Rich (Annonaceae), *Pinusoccidentalis* Sw. (Pinaceae), and *Sloanea* L. (Elaeocarpaceae).

There are two documented populations of *Gesneriatuberifera*, and both are south of the Sierra de Bahoruco National Park. Thus, there are no known populations of *G.tuberifera* within the Sierra de Bahoruco National Park or other protected areas. The two populations of *G.tuberifera* are within relatively easy access to major roads. The population from the type locality (Pedernales) includes approximately 100 individuals, covering a vertical limestone outcrop. The population from Enríquillo (Barahona) was more limited, with fewer than 50 individuals. The area of occupancy (AOO) was calculated as 12 km^2^. Following the IUCN Red List Categories and Criteria (IUCN 2012) and guidelines of the IUCN Standards and Petitions Committee (2022), *Gesneriatuberifera* is preliminarily assessed as Endangered (EN), which is supported by a restricted population of less than 250 mature individuals (D).

#### Comments.

**Figure 2.**
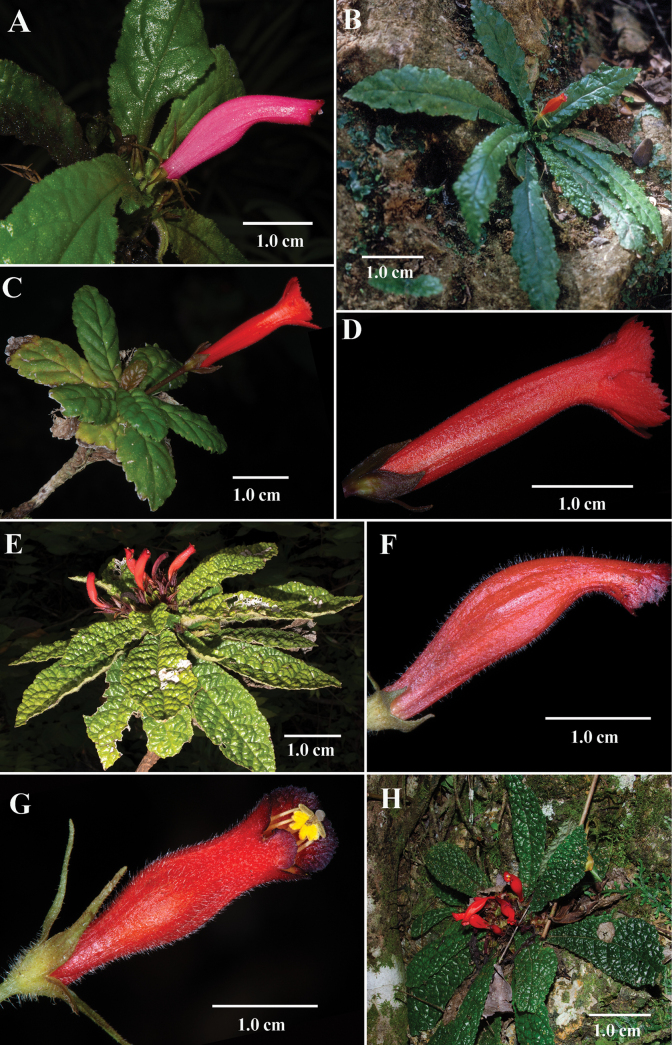
*Gesneria* taxa with lithophytic habits and elongate tubular red corollas **A***Gesneriaacaulis* L. **B***Gesneriachristii* Urb. **C, D***Gesneriareticulata* (Griseb.) Urb. **E, F***Gesnerialibanensis* Linden ex C. Morren **G***Gesneriapedicellaris* Alain **H***Gesneriapurpurascens* Urb. **(A***J.L. Clark 14532***B***T. Talpey s.n.***C, D***J.L. Clark 17420***E, F***J.L. Clark 15987***G***J.L. Clark 17934***H***J.L. Clark 12769*). Photos by John L. Clark

Most *Gesneria* taxa are multibranched terrestrial or lithophytic shrubs, 1–2 m tall. There are fewer than 10 species of *Gesneria* with a lithophytic habit with leaves in a rosette. Rosette-forming here is broadly defined to include taxa where there is a basal rosette of leaves without a developed stem (Fig. 1B, E), a growth habit typical of many herbaceous annuals. In addition, rosette-forming can also describe perennial elongate shoots that might reach a length of 30 cm where an apical rosette of leaves is produced (Fig. 2E). It is common in older individuals of lithophytic *Gesneria* taxa to develop unbranched elongate shoots that subtend rosettes of leaves. In contrast, lithophytic shrubs are often branched, reach 2 meters in height, but never form rosettes or clusters of leaves. *Gesneriatuberifera* includes individuals with basal rosettes of leaves (Fig. 1E) and individuals with unbranched elongate shoots (ca. 30 cm long) subtending a terminal rosette of leaves.

A recent phylogenetic study by Joly et al. (2018) strongly supported the presence of several clades of shrubs (terrestrial or lithophytic) with hummingbird specialist pollination syndromes, but only one clade (fig. 6 in Joly et al. 2018) correlates with obligate lithophytes with leaves in a rosette. Many of the lithophytic rosette taxa are similar to *Gesneriatuberifera*, such as *G.acaulis* L. (Fig. 2B) from Jamaica, *G.cuneifolia* (DC.) Fritsch from Puerto Rico, *G.pedicellaris* Alain (Fig. 2G) from Dominican Republic, *G.purpurascens* Urb. (Fig. 2H) from Cuba, *G.reticulata* (Griseb.) Urb. (Fig. 2C, D) from Puerto Rico and Hispaniola, and *G.yamuriensis* Britton & P.Wilson from Cuba. Other *Gesneria* lithophytes with leaves in a rosette that were not represented in the phylogeny in Joly et al. (2018) include *Gesneriachristii* Urb. (Fig. 2A) from Hispaniola and *G.libanensis* Linden ex C. Morren (Fig. 2E, F) from Cuba. *Gesneriatuberifera* differs from all other lithophytes with a rosette of leaves by the presence of tubers (Fig. 1C) and tubular red corollas with yellow lobes (Fig. 1A). In contrast, most lithophytic *Gesneria* with rosette leaves have uniformly red tubular corollas (Fig. 2) without contrasting coloration on the lobes.

**Figure 3.**
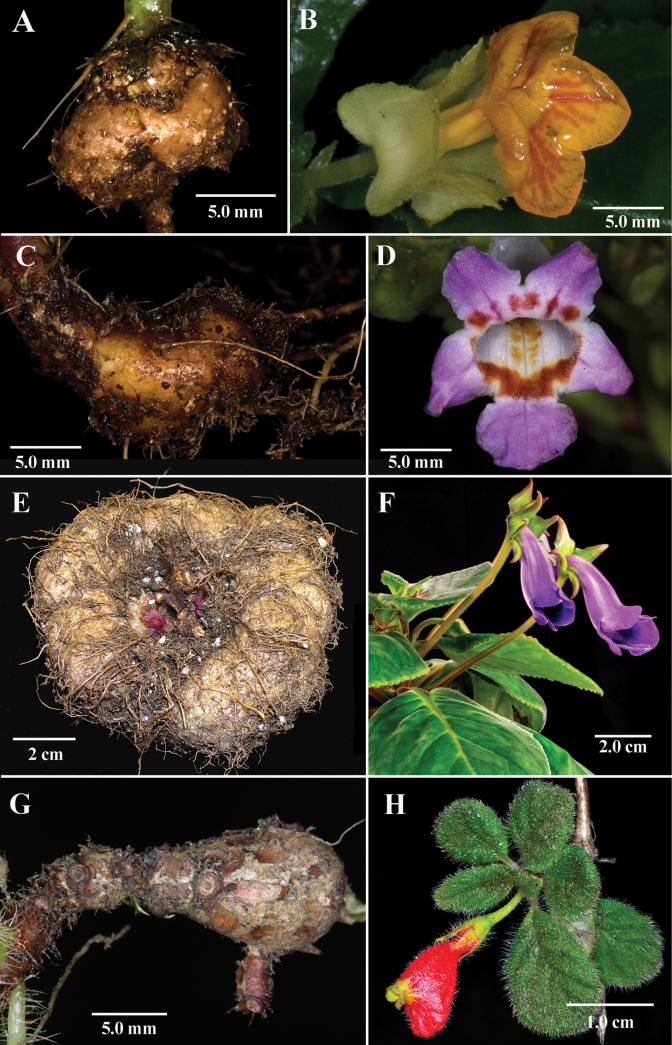
Gesneriaceae taxa with tubers **A, B***Chrysothemisfriedrichsthaliana* (Hanst.) H.E.Moore **C, D***Trichodrymoniapedunculata* (L.E.Skog) M.M.Mora & J.L.Clark **E, F***Sinningiaspeciosa* (Lodd.) Hiern ‘Niemeyer’ **G, H***Pachycaulosnummularia* (Hanst.) J.L.Clark & J.F.Smith (**A, B***J.L. Clark 12699***C, D***J.L. Clark 12680***E, F** D. *Zaitlin s.n*. **G, H***J.L. Clark 16357*). Photos **A, B, C** and **D** by John L. Clark and photos **E, F** by David Zaitlin.

The presence of tubers is relatively rare in Gesneriaceae. The only large genus (65+ species) where tubers are common is *Sinningia* Nees mainly from Brazil, which includes the commonly cultivated species *Sinningiaspeciosa* (Lodd.) Hiern (Fig. 3E, F). Several small genera have tubers such as the monotypic genus *Lembocarpus* Leeuwenb. and *Rhoogeton* Leeuwenb. (2 species). Tubers are also present in *Pachycaulos*, a genus that was recently expanded (Clark et al. 2023) to include two species, *Pachycauloshuancabambae* J.L.Clark & Moonlight and *P.nummularia* (Hanst.) J.L.Clark & J.F.Smith (Fig. 3G, H). The tribe Sphaerorrhizeae was established by Roalson and Boggan (Roalson et al. 2005) to accommodate a small clade of four species that are partly characterized by “stringy” rhizomes with tuber-like swellings. Other examples of tubers in Gesneriaceae are limited to single taxa within genera that are not usually tuberous. For example, *Chrysothemisfriedrichsthaliana* (Hanst.) H.E. Moore forms tubers (Fig. 2A, B), but their presence is inconsistent. Even when actively looking for tubers in wild populations of *Chrysothemisfriedrichsthaliana*, their presence is sometimes completely absent or present in only a few individuals. Tubers are mostly absent in *Trichodrymonia*, but one exception is *Trichodrymoniapedunculata* (L.E.Skog) M.M.Mora & J.L.Clark where tubers are consistently present in cultivation and in the wild (Fig. 3C, D) in eastern Panama. The presence of tubers in *Gesneriatuberifera* is the first documented example of this unusual character in the genus and it represents the only member of Gesneriaceae with tubers from the Caribbean.

The presence of tubers is mostly unknown in the Old World members of Gesneriaceae. The recently described monotypic genus, whose single species, *Michaelmoelleriavietnamensis* F.Wen, Z.B.Xin & T.V.Do, was not reported to have tubers when it was described from field collections (Wen et al. 2020), but horticulturists have noticed tubers in cultivation (D. Zaitlin, pers. comm.). Weber (2004) provides a comprehensive survey on the range of morphological features documented throughout Gesneriaceae, and reports that tubers are confined to the New World Gesneriaceae. Thus, the presence of tubers in *Michaelmoelleria* F.Wen, Y.G.Wei & T.V.Do is the first and only known species of Old World Gesneriaceae with tubers.

#### Additional specimens examined.

**Dominican Republic. Barahona**: Sierra de Bahoruco, road from the coastal town of Enriquillo towards the community Blanco, area known locally as El Fondo Farallon, adjacent to Río Fondo, 17°57'39.04"N, 71°13'50.16"W, 407 m, 28 Jan 2023, *J.L. Clark et al. 17312* (FLAS, JBSD, MO, MT, NY, SEL, US); Sierra de Bahoruco, Municipio Enríquillo, seccion Los Blancos, paraje El Fondo, subiendo hacia loma Materesa, 600 m, 11 Jun 2009, *B. Peguero et al. 4717* (JBSD). **Pedernales**: Sierra de Bahoruco, road Cabo Rojo–Las Mercedes, Finca de Isabel, 18°06'50.09"N, 71°37'10.14"W, 738 m, 26 Jan 2023, *J.L. Clark & T. Clase 17284* (FLAS, JBSD, MO, MT, NY, SEL, US); Sierra de Bahoruco, sección Las Mercedes, Aceitillar, subiendo en la carretera Cabo Rojo, 18°6'8.04"N, 71°37'14.3"W, 400–500 m, 10 Jun 2007, *T. Clase et al. 4526* (JBSD); Sierra de Bahoruco, km 26 Norte desde el Puerto de Cabo Rojo (de la Alcoa Exploration Company) en el camino minero a Las Mercdes y Aceitillar, 18°06'N, 71°36'W, 610 m, 16 Feb 1982, *T. Zanoni*, *M. Mejía*, *J. Pimentel & J.T. Mickel 19068* (JBSD, NY); Sierra de Bahoruco, entre los kms. 25–28 de la Carretera Puerto de Cabo Rojo hacia Aceitillar, 18°06'N, 71°37'W, 820 m, 1 Aug 1984, *M. Mejía*, *J. Pimentel & R. García 1076* (JBSD).

## Supplementary Material

XML Treatment for Gesneria tuberifera

## ﻿Additional information

### Author ORCIDs

John L. Clark https://orcid.org/0000-0002-1414-6380

### Data availability

All of the data that support the findings of this study are available in the main text.
